# Genome-wide discovery of multiple sclerosis genetic risk variant allelic regulatory activity

**DOI:** 10.1093/g3journal/jkaf192

**Published:** 2025-08-21

**Authors:** Marissa Granitto, Lois Parks, Molly S Shook, Carmy Forney, Xiaoting Chen, Lee E Edsall, Omer A Donmez, Sreeja Parameswaran, Kristen S Fisher, Aram Zabeti, Lucinda P Lawson, Matthew T Weirauch, Leah C Kottyan

**Affiliations:** Center for Autoimmune Genomics and Etiology, Cincinnati Children's Hospital Medical Center, Cincinnati, OH 45229, United States; Development, Stem Cells & Regenerative Medicine Graduate Program, University of Cincinnati, Cincinnati, OH 45229, United States; Division of Allergy and Immunology, Cincinnati Children's Hospital Medical Center, Cincinnati, OH 45229, United States; Center for Autoimmune Genomics and Etiology, Cincinnati Children's Hospital Medical Center, Cincinnati, OH 45229, United States; Division of Allergy and Immunology, Cincinnati Children's Hospital Medical Center, Cincinnati, OH 45229, United States; Immunology Graduate Program, University of Cincinnati, Cincinnati, OH 45229, United States; Center for Autoimmune Genomics and Etiology, Cincinnati Children's Hospital Medical Center, Cincinnati, OH 45229, United States; Division of Allergy and Immunology, Cincinnati Children's Hospital Medical Center, Cincinnati, OH 45229, United States; Center for Autoimmune Genomics and Etiology, Cincinnati Children's Hospital Medical Center, Cincinnati, OH 45229, United States; Division of Allergy and Immunology, Cincinnati Children's Hospital Medical Center, Cincinnati, OH 45229, United States; Center for Autoimmune Genomics and Etiology, Cincinnati Children's Hospital Medical Center, Cincinnati, OH 45229, United States; Division of Allergy and Immunology, Cincinnati Children's Hospital Medical Center, Cincinnati, OH 45229, United States; Center for Autoimmune Genomics and Etiology, Cincinnati Children's Hospital Medical Center, Cincinnati, OH 45229, United States; Division of Allergy and Immunology, Cincinnati Children's Hospital Medical Center, Cincinnati, OH 45229, United States; Center for Autoimmune Genomics and Etiology, Cincinnati Children's Hospital Medical Center, Cincinnati, OH 45229, United States; Division of Allergy and Immunology, Cincinnati Children's Hospital Medical Center, Cincinnati, OH 45229, United States; Center for Autoimmune Genomics and Etiology, Cincinnati Children's Hospital Medical Center, Cincinnati, OH 45229, United States; Division of Allergy and Immunology, Cincinnati Children's Hospital Medical Center, Cincinnati, OH 45229, United States; Department of Pediatrics, Division of Neurology and Developmental Neuroscience, Baylor College of Medicine, Houston, TX 77030, United States; Wadell Center for Multiple Sclerosis, College of Medicine, University of Cincinnati, Cincinnati, OH 45220, United States; Center for Autoimmune Genomics and Etiology, Cincinnati Children's Hospital Medical Center, Cincinnati, OH 45229, United States; Division of Allergy and Immunology, Cincinnati Children's Hospital Medical Center, Cincinnati, OH 45229, United States; Center for Autoimmune Genomics and Etiology, Cincinnati Children's Hospital Medical Center, Cincinnati, OH 45229, United States; Division of Allergy and Immunology, Cincinnati Children's Hospital Medical Center, Cincinnati, OH 45229, United States; Division of Biomedical Informatics, Cincinnati Children's Hospital Medical Center, Cincinnati, OH 45229, United States; Division of Developmental Biology, Cincinnati Children's Hospital Medical Center, Cincinnati, OH 45229, United States; Department of Pediatrics, University of Cincinnati, College of Medicine, Cincinnati, OH 45229, United States; Center for Autoimmune Genomics and Etiology, Cincinnati Children's Hospital Medical Center, Cincinnati, OH 45229, United States; Division of Allergy and Immunology, Cincinnati Children's Hospital Medical Center, Cincinnati, OH 45229, United States; Department of Pediatrics, University of Cincinnati, College of Medicine, Cincinnati, OH 45229, United States; Division of Immunobiology, Cincinnati Children's Hospital Medical Center, Cincinnati, OH 45229, United States

**Keywords:** multiple sclerosis, MS, massively parallel reporter assay, B cells

## Abstract

Multiple sclerosis is an immune-mediated demyelinating disease of the central nervous system with a complex etiology involving environmental and genetic factors. Numerous genetic risk loci for multiple sclerosis have been nominated through genome-wide association studies, with most associated variants residing in noncoding regions. However, further work is needed to understand how genetic variation contributes to disease-related alterations to gene expression. Here, we use Massively Parallel Reporter Assays to identify genetic risk variants with genotype-dependent enhancing or silencing activity within a set of 14,275 variants distributed among multiple sclerosis risk loci that have reached genome-wide or suggestive significance. We applied our Massively Parallel Reporter Assay library to Epstein-Barr-virus–transformed B cell lines derived from two patients with multiple sclerosis, as well as the ENCODE Tier 1 cell line GM12878. In total, our approach discovered 150 allelic enhancing variants and 286 allelic silencing variants, collectively representing 83 independent multiple sclerosis risk loci. Our systematic, genome-scale approach implicates potentially causal genotype-dependent gene regulatory mechanisms for over a third of the known multiple sclerosis risk loci, providing a unique resource for the discovery of the genetic mechanisms underlying this chronic inflammatory disease.

## Introduction

Multiple sclerosis (MS) is an immune-mediated demyelinating disease of the central nervous system (CNS) (Filippi et al. 2018). Global prevalence is increasing, affecting over 2.9 million people worldwide (Walton et al. 2020). The exact cause of MS is unknown, but genetic factors account for up to 50% of disease risk, with the unaccounted risk being attributed to environmental factors including Epstein-Barr virus (EBV) infection (International Multiple Sclerosis Genetics 2019; Olsson et al. 2017).

Genome-wide association studies (GWASs) have identified 233 independent risk loci for MS. There are 201 independent risk loci outside of the major histocompatibility complex (MHC) and 32 independent risk loci inside the MHC region (International Multiple Sclerosis Genetics 2019). Most risk variants are common (minor allele frequency > 1%) and have small effect sizes (odds ratio [OR] of 1.05 to 1.2). Only one known risk variant, which encodes HLA-DRB*1501, has a moderate effect size (OR ∼3) (Hollenbach and Oksenberg 2015).

Despite over 200 independent genetic associations, the genotype-dependent biology impacted by these variants is only known for a handful of loci (Putscher et al. 2022). Linkage disequilibrium (LD) within risk loci complicates efforts to identify the specific variants that mechanistically contribute to MS. Variants are enriched in noncoding regions of the genome, including enhancers and promoters, suggesting that transcriptional dysregulation may be a key to MS pathogenesis (Maurano et al. 2012). In this study, we thus sought to systematically identify potentially causal variants underlying the genetic associations at all known MS-associated risk loci.

The majority of known MS risk genes are immune-related rather than CNS-related. These genes are mostly expressed in B cells, T cells, and/or monocytes (International Multiple Sclerosis Genetics 2019). B cells have been implicated in the etiology of MS based on numerous lines of evidence. Epidemiologically, EBV infection and reactivation is strongly implicated in the development and pathogenesis of MS, and B cells serve as the cell type infected by EBV and the reservoir for latent EBV (DeLorenze et al. 2006; Abrahamyan et al. 2020; Bjornevik et al. 2022; Lanz et al. 2022; Robinson and Steinman 2022). Clinically, B cells serve as antigen-presenting cells and produce the autoreactive antibodies that lead to inflammation in the CNS, where they cause local inflammation after binding antigens such as myelin oligodendrocyte glycoprotein, GAGA4, and KIR4.1 (DeMarshall et al. 2017; Ahn et al. 2021; Rastogi et al. 2022). Therapeutically, anti-B cell therapies have become a frontline, highly efficacious approach to treating MS and suspected MS (Stathopoulos and Dalakas 2022). The risk loci for MS are highly enriched for genomic loci with accessible chromatin in B cells that is bound by inflammatory effector molecules such as NFkB, AP-1, and EBV-encoded EBNA2 (Harley et al. 2018; Hong et al. 2021; Ma et al. 2023; Viel et al. 2024).

In this study, we use Massively Parallel Reporter Assays (MPRAs) to systematically assess the effects of MS risk variants on transcriptional regulation in B cells. Our MPRA library includes all published MS genetic tag variants (Buniello et al. 2019) as well as all variants with strong LD (*r*^2^ > 0.8) to these tag variants. We modified an established transient MPRA protocol (Tewhey et al. 2016; Ulirsch et al. 2016; Lu et al. 2021; Shook et al. 2024) to use a respiratory syncytial virus (RSV) promoter of moderate strength instead of a standard minimal promoter. This strategy enables us to simultaneously identify variants with allelic enhancing activity as well as variants with allelic silencing activity. In addition to the commonly used and commercially available ENCODE Tier 1 GM12878 cell line, we performed our assays in two MS patient-derived B cell lines. Our results show allele-dependent regulatory activity at 83 out of the 217 tested independent MS risk loci. Both allelic enhancers and allelic silencers are connected to genes involved in antigen presentation, but allelic silencers are uniquely enriched for pathways related to transcription regulation and chromatin remodeling. Collectively, these results nominate putative causal variants that result in genotype-dependent transcriptional regulation, providing an important resource for understanding the contribution of genetic risk variants to MS.

## Methods

### Ethics statement

This study was approved by the Cincinnati Children's Hospital Institutional Regulatory Board (IRB #2017-0430) in March, 2017. Two individuals with clinician-diagnosed MS were recruited from the Waddell Center for Multiple Sclerosis at the University of Cincinnati or through a study advertisement sent out to Cincinnati Children's Hospital employees (see below for clinical details). Both participants signed informed consent forms prior to participation.

### Variant selection and DNA sequence generation for MPRA

**Fig. 1.**
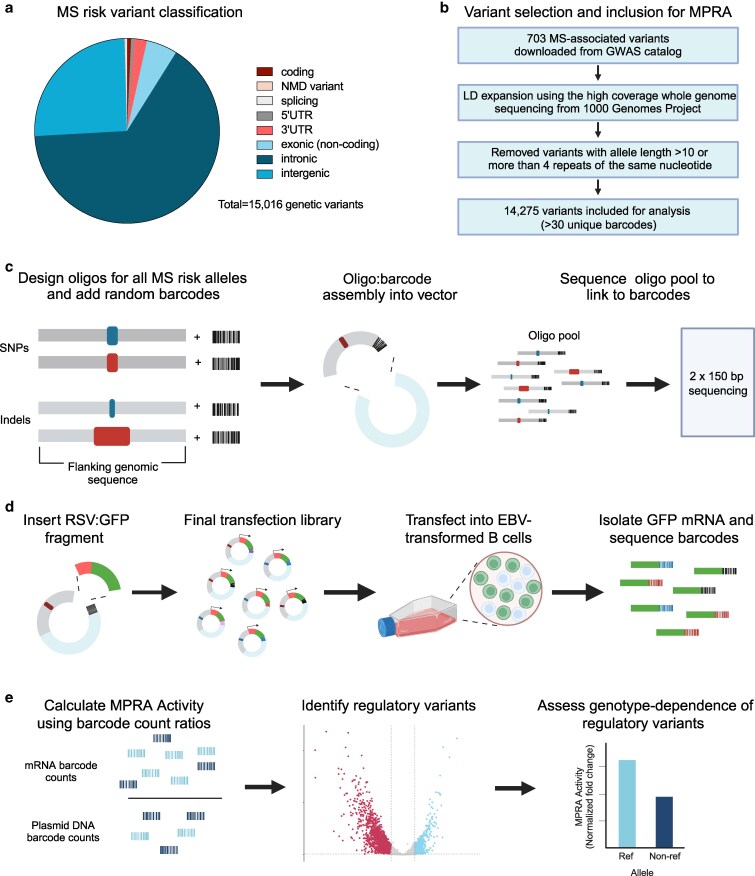
Scheme for the identification of regulatory variants using MPRAs. a) Variant classification of all MS-associated risk variants in the MPRA. b) Variant selection method for MPRA construction and analysis. c) Schematic of MPRA oligo (allele) design and assembly. d) Schematic of MPRA library construction and experiments. e) Schematic of MPRA analytical framework.

All MS-associated genetic risk loci published through August 2021 were included in this study, spanning coding and noncoding regions (Fig. 1a). These 703 variants were downloaded from the GWAS Catalogue (Buniello et al. 2019) (Fig. 1b, Supplementary Table 1). We included all variants reaching genome-wide (*P* < 5 × 10^−8^) and suggestive significance (5 × 10^−8^ < *P* < 1 × 10^−5^). GWAS tag variants are those that are directly reported for each locus; however, risk loci contain multiple other variants in strong LD with one another. Each of these variants is potentially causal and could have allelic regulatory activity. Tag variants were thus used for LD expansion (*r*^2^ > 0.8) based on 1,000 Genomes Data (Byrska-Bishop et al. 2022), using the ancestries of the initial genetic association and using the PLINK (v1.90b) software package (Supplementary Table 2, Fig. 1b) (Purcell et al. 2007). We removed those insertion/deletion variants with an allele length greater than 10 as well as those variants with more than four repeats of the same nucleotide. Unmappable variants were discarded. For single nucleotide polymorphisms, we obtained 170 base pairs of hg38-flanking DNA sequences, with the variant located in the center (84 bps upstream and 85 bps downstream of the variant), with identical flanking genomic sequences across each allele. For indels, we designed the flanking sequences to ensure that each oligo was 170 bps. Adapters (15 bps) were added to each sequence at each end (5′-ACTGGCCGCTTGACG—[170 bp oligo]—CACTGCGGCTCCTGC-3′) to make a 200 bp DNA sequence (Fig. 1c). The final list of all LD-expanded variants can be found in Supplementary Table 2. For all resulting sequences, we created a forward and reverse complement sequence to compensate for possible DNA synthesis errors. A total of 82,622 oligos were ordered from Twist Bioscience (Supplementary Table 3).

### Library construction

For assembly of the MPRA library, we followed the procedure originally described by Tewhey et al. (2016) with minor modifications as described in Lu et al 2021 (Tewhey et al. 2016; Lu et al. 2021 ; Shook et al. 2024) (Fig. 1d). Briefly, we labeled oligonucleotides with degenerate 20-mer barcode sequences using PCR, then cloned them into a backbone vector to create a library. An RSV + eGFP fragment was amplified from pRSC-gfp-hAIM2 (Addgene #51666) through 8× PCR with a 50 µL system, each containing 1 ng plasmid, 25 μL NEBNext® Ultra™ II Q5® Master Mix, 0.5 μM 200-MPRA_v3_GFP_Fusion_v2_F, and 0.5 μM 201-MPRA_v3_GFP_Fusion_v2_R. PCR was performed under the following conditions: 98 °C for 2 min, 20 cycles of (98 °C for 10 s, 60 °C for 15 s, 72 °C for 45 s), 72 °C for 5 min. The amplified product was purified and then inserted into the AsiSI- digested backbone library through Gibson assembly at 50 °C for 1.5 h to create the transfection library. The resulting library was re-digested by RecBCD and AsiSI, purified, and then transformed into *E. coli* through electroporation (2 kV, 200 ohm, 25 μF). Transformed *E. coli* was cultured in 5 L of LB Broth buffer supplemented with 100 μg/mL of carbenicillin at 37 °C for 12–16 h. The plasmid was then extracted using the QIAGEN Endo-free Plasmid Giga Kit (cat # 12391). For oligo and barcode association, the plasmid was purified, molar pooled, and sequenced using 2 × 150 bp on an Illumina NovaSeq 6000. All primer sequences used in this study can be found in Supplementary Table 4.

### Oligo and barcode association

Paired-end, 150 bp reads were first quality filtered using Trimmomatic (v.0.38) (flags: PE -phred33, LEADING:25, TRAILING:25, MINLEN:80) (Bolger et al. 2014). Read 1 was separated into the 20 bp barcode region and the oligo-matching region. The trimmed oligo-matching regions of read 1 and read 2 were mapped back to the synthesized oligo sequences using Bowtie2 (v.2.3.4.1) (flags: -X 250, -very-sensitive, -p 16) (Langmead and Salzberg 2012). Barcodes were then associated with the oligo sequences using the read ID. The backbone library has a median of 239 unique barcodes per oligo (Supplementary Fig. 1).

### Generation of EBV-transformed B cell lines

Two EBV-transformed B cell lines were derived from individuals with MS. The cell line MS-1 was derived from an individual being treated with dimethyl fumarate. The cell line MS-2 was derived from an individual not being treated with any disease-modifying therapies. Immediately after sample collection, peripheral blood mononuclear cells (PBMCs) were isolated from blood samples by Ficoll density gradient centrifugation. CD19+ primary B cells were isolated from PBMCs using a Miltenyi B cell isolation kit (Miltenyi Biotec #130-091-151). Wild-type B95.8 EBV was prepared from B95-8 cell supernatants cultured in 10% fetal bovine serum (FBS)-supplemented RPMI-1640 medium for 2 weeks. Viral suspension was filtered twice with 0.45 µm Millipore filters. The concentrated viral stocks were stored at −80 °C. Primary B cells were infected with 1 mL of viral stock based on infection optimization assays and incubated for 3 h for virus adsorption. Within the first month of cell culture, we confirmed EBV infection by morphological changes and clumping of the cells. Cells were collected for experiments after at least four passages.

### MPRA transfection

GM12878 and the two MS-derived EBV-transformed B cell lines (MS-1 and MS-2) were grown in RPMI medium supplemented with 10% FBS, 100 µg/mL normocin, and 1× antibiotic–antimycotic. Cells were seeded at a density of 5 × 10^5^ cells/mL the day before transfection. For each cell line, we transfected five replicates and used a total of 7 × 10^7^ cells per replicate. Electroporation was performed using the Neon transfection system in 100 µL tips with 3 pulses of 1,200 V, 20 ms each. After transfection, cells were recovered in prewarmed media supplemented with 10% FBS at a density of 1.5 × 10^6^ cells/mL for 24 h. Cells were then collected for preparation of the sequencing libraries.

### Sequencing library for barcode counting

Total RNA of transfected cells was extracted by the RNeasy Midi Kit (Qiagen #74104) following the manufacturer's instructions (Fig. 1d). Extracted RNA was subjected to DNase treatment in a 750 μL system with 5 μL Turbo DNase and 75 μL Turbo DNase Buffer at 37 °C for 1 h. 7.5 μL 10% SDS and 75 μL 0.5 M EDTA were added to stop DNase with 5 min of incubation at 75 °C. The whole volume was used for eGFP probe hybridization in a 3.6 mL system, with 900 μl 20× SSC buffer, 1,800 μL Formamide and 2 μL of each 100 μM Biotin-labeled GFP probes #1-3. The probe hybridization was performed through incubation at 65 °C for 2.5 h. 400 μL Dynabeads™ MyOne™ Streptavidin C1 was prepared according to the manufacturer's instructions. The beads were suspended in 500 μL 20× SSC buffer and incubated with the above probe hybridization reaction at room temperature for 15 min. Beads were then collected on a magnet and washed with 1× SSC buffer once, and 0.1× SSC buffer twice. *eGFP* mRNA was eluted first through adding 25 μL ddH_2_O, heating at 70 °C for 2 min and collecting on a magnet, then adding another 25 μL ddH_2_O, heating at 80 °C for 2 min and collecting on a magnet. A final DNase treatment was performed on all of the purified RNA in a 30 μL system containing 0.5 μL Turbo DNase and 3 μL Turbo DNase Buffer at 37 °C for 1 h. 0.5 μL 10% SDS was added to halt DNase reaction. Eluted mRNA was purified through RNA Clean SPRI Beads. mRNA was reverse transcribed to cDNA using SuperScript™ IV First-Strand Synthesis System with the gene-specific primer MPRA_v3_Amp2Sc_R, following the manufacturer's instructions. cDNA was then used for building sequencing libraries following the Tag-seq Library Construction section in Tewhey et al. (2016). In brief, 1 μL of cDNA and samples were used to estimate the relative concentration of *eGFP* in the 10 μL system containing 5 μL NEBNext® Ultra™ II Q5® Master Mix, 0.6 μL SYBR green I diluted 1:1,000 (Life Technologies, S7563), and 0.5 μM TruSeq_Universal_Adapter_P5 and MPRA_V3_Illumina_GFP_F. PCR was performed under the following conditions: 95 °C for 20 s, 40 cycles of (95 °C for 20 s, 65 °C for 20 s, 72 °C for 30 s), 72 °C for 2 min. A total of two PCRs were needed for building the sequencing library. The first PCR was performed with 10 μL of normalized samples in the 50 μL system containing 25 μL NEBNext® Ultra™ II Q5® Master Mix, 0.5 μM TruSeq_Universal_Adapter_P5, and MPRA_V3_Illumina_GFP_F. PCR was performed under the following conditions: 95 °C for 20 s, corresponding cycles of (95 °C for 20 s, 65 °C for 20 s, 72 °C for 30 s), 72 °C for 2 min. The product was then purified, and indices were added through a 100 μl system containing all purified product, 50 μl NEBNext® Ultra™ II Q5® Master Mix, 0.5 μM TruSeq_Universal_Adapter_P5, and index primer. PCR was performed as above, except using only 6 cycles. The sample replicates were purified, molar pooled, and sequenced 1 × 150 bp on Illumina NovaSeq 6000 or Illumina NovaSeq X Plus.

### Barcode quantification

Single-end 100 bp reads were first quality filtered using Trimmomatic (v.0.38) (flags: SE -phred33, LEADING:3, TRAILING:3, MINLEN:70). Each read was then separated into the 20 bp barcode region and the constant region. The trimmed constant regions of the reads were mapped back to the constant region within the *eGFP* 3′ UTR using Bowtie2 (v.2.3.4.1) (flags: -very-sensitive, -p 16). Only reads with Levenshtein distance of 4 or less within the constant region and perfect matches to the two bases directly adjacent to the barcode were kept. Barcodes were then associated with the retained reads using the read ID.

#### Regulatory variant identification

All barcodes were summarized at the oligo (allele) level. Oligos with 30 or more unique barcodes from the plasmid replicates were included for downstream analysis. As an important quality control step, we compared the normalized signals between replicates in each cell type (Supplementary Fig. 2). Barcode count totals for each oligo, including all MS variants and the 20 control variants, were passed into DESeq2 (v1.42.1) in R (v4.3.2) to estimate the fold change and significance between plasmid controls and the experimental replicates (Love et al. 2014). The control variants include 20 variants that were examined in a previous MPRA experiment that was also performed in the GM12878 cell line (Lu et al. 2021). Supplementary Table 5 reports robust agreement between the two types of promoters (67% agreement in enhancer detection), despite the use of different MPRA promoter sequences (minimal promoter vs RSV promoter). A Benjamini–Hochberg false discovery rate (FDR) adjusted *P*-value of <0.05 was required for significance. Only significant alleles with greater than a 1.2-fold change were identified as enhancer alleles. A variant was identified as an enhancer variant if any allele of the variant was an enhancer allele. Significant alleles with more than a 1.2-fold decrease in expression compared to the plasmid controls were identified as silencer alleles. A variant was considered a silencer if any allele of the variant was a silencer allele. One regulatory variant, “rs62405579_Ref_C”, was removed from downstream analysis because it had opposite effects in two cell lines.

#### Allelic regulatory variant identification

Only regulatory variants (enhancers or silencers) were considered for allelic analysis. The barcode counts from every allele of each enhancer/silencer variant were used for calculating *P*-values. *P*-values were calculated by comparing the log2 ratios of the nonreference allele to the reference allele, normalized by plasmid controls, using Student's *t*-test. *P*-values were adjusted with the Benjamini–Hochberg FDR-based procedure. A corrected *P*-value of <0.05 was required for significance. Only significant alleles with a 20%-fold change or greater were classified as allelic. We used the R package mpraprofiler for performing this analysis, which is available on our GitHub page (https://github.com/WeirauchLab/mpraprofiler).

### Pathway enrichment analysis

Variants were annotated with all SNP-gene associations from the eQTL Catalogue (Kerimov et al. 2021) that were significant at nominal *P*-value <0.05 in lymphoblastoid cell lines (LCLs), which are EBV-immortalized B cells. Allelic regulatory variants were only annotated for SNP-gene associations in the direction of the allelic effect. Pathway analysis was performed using Enrichr (Chen et al. 2013; Kuleshov et al. 2016).

### Functional genomics dataset enrichment analysis with RELI

499 ChIP-seq datasets from EBV-immortalized B cell lines (LCLs) were obtained from the Gene Expression Omnibus (GEO) using custom scripts that searched for ChIP-seq experiments followed by manual annotation. The Sequence Read Archive files obtained from GEO were analyzed using an automated peak calling pipeline using MACS2 as described in Lu et al. (2021). We used the RELI (v.0.9) algorithm (Harley et al. 2018) to identify genomic features (TF binding events and histone marks identified as ChIP-seq peaks) that coincide with regulatory and allelic regulatory variants, also as described in Lu et al. (2021). All oligos contained in the MPRA transfection library were used as a “negative set” for comparison to the “positive” (input) set, and *P*-values were estimated using RELI, based on the default 2,000 iterations of random sampling for estimating the significance of the observed number of input regions that overlap each ChIP-seq dataset.

## Results

### Design and application of an MPRA to assess MS genetic risk variants

We designed an MPRA strategy to systematically investigate MS-associated genetic variants for effects on transcriptional activity (Fig. 1—see Methods). Similar to what is observed for other diseases (Maurano et al. 2012), over 95% of the MS-associated variants contained in our MPRA are located in noncoding regions of the genome (Fig. 1a). After variant selection and quality control filtering, we included a total of 14,275 variants in our downstream analyses, with an average of 2.7 alleles per variant (Fig. 1b, Supplementary Table 5).

### Enhancing and silencing regulatory activity at MS risk variants

To assess the regulatory activity of all MS-associated alleles across multiple MS-relevant cell types, we transfected five replicates of our library into EBV-transformed B cells from two patients with MS and the ENCODE tier 1 EBV-transformed B cell line GM12878. Measurement of enhancing and silencing activity for each oligo entailed isolation and sequencing of the barcoded *eGFP* mRNA, followed by normalization of this RNA barcode signal to the plasmid DNA library (see Methods). Each of the five replicates from each of the three EBV-transformed B cells were of high quality and showed excellent reproducibility. In particular, experiments clustered by cell type, with large separation from controls (Supplementary Fig. 2a). Likewise, experimental replicates were highly correlated with one another, but not with controls (Supplementary Fig. 2b-d).

**Fig. 2.**
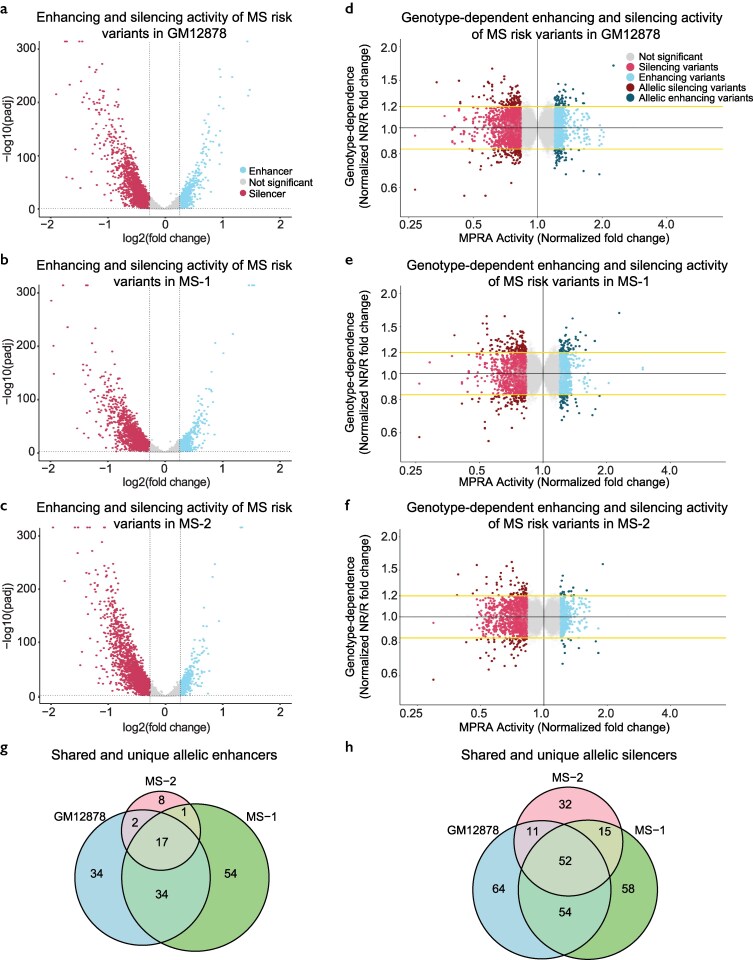
Identification of MS regulatory variants and allelic regulatory variants. a to c) Enhancer and silencer variant identification in each of the three cell lines. The *x*-axis denotes the fold-change difference in oligo expression in the replicates compared to the plasmid controls. Vertical dashed lines indicate a fold-change threshold of 1.2. The *y*-axis denotes the significance. Horizontal dashed lines indicate an adjusted *P*-value significance threshold of 0.05. Results are shown for GM12878 (a), as well as cell lines derived from two patients with multiple sclerosis: MS-1 (b), and MS-2 (c). d to f). Allelic regulatory variant identification. Genotype-dependence (*y*-axis) was calculated as the normalized fold change in MPRA activity between the nonreference (NR) and reference (R) alleles for each variant. MPRA activity (*x*-axis) is the highest fold change in MPRA activity relative to controls for any allele of a variant. Dark blue dots represent allelic enhancer variants with a significant difference in MPRA activity between any two alleles (p_FDR_ < 0.05) and at least a 20% difference in MPRA activity between alleles. Dark red dots represent allelic silencer variants with a significant difference in MPRA activity between any two alleles (p_FDR_ < 0.05) and at least a 20% difference in MPRA activity between alleles. Results are shown for GM12878 (d), MS-1 (e), and MS-2 (f). g) Quantification of the number of shared and unique allelic enhancers in GM12878, MS-1, and MS-2. h) Shared and unique allelic silencers in GM12878, MS-1, and MS-2.

We next identified risk variants with enhancing or silencing activity. A variant was considered an enhancer or silencer variant if it had statistically significant enhancing/silencing activity (p_adj_ < 0.05) and at least a 1.2-fold increase/decrease in transcriptional activity compared to the barcode counts in the plasmid controls (see Supplementary Fig. 3 for schematic). In each cell line, we found more silencing variants than enhancing variants. In GM12878 we identified 788 enhancing variants and 1,542 silencing variants (Fig. 2a). In MS-1, we identified 836 enhancing variants and 1,683 silencing variants (Fig. 2b). In MS-2, we identified 380 enhancing variants and 1,373 silencing variants (Fig. 2c). There was high overlap between the three cell lines, especially for variants with silencing activity (Supplementary Fig. 4). Across all cellular contexts, we identified 1,141 unique variants (8%) with enhancing activity and 2,122 unique variants (15%) with silencing activity (Supplementary Fig. 4, Supplementary Table 5). To better understand the potential functions of these variants, we searched for functional genomics features enriched within each class of regulatory variants using the RELI algorithm (Harley et al. 2018). As expected, the enhancer variants were much more strongly enriched for the active histone mark H3K27ac than the silencer variants (Supplementary Fig. 5, Supplementary Table 6).

This dataset of significant enhancing and silencing variants was next assessed for allelic regulatory activity. We defined allelic regulatory variants as enhancer or silencer variants that have significant genotype-dependent regulatory activity and also have at least a 20% difference in expression between alleles (FDR-adjusted *P*-value < 0.05, Student's *t*-test, see Supplementary Fig. 3 for schematic). Expression was compared between the reference and nonreference alleles for each variant (provided in Supplementary Table 2), with complete results available in Supplementary Table 7. With these definitions, we identified 87 allelic enhancing and 181 allelic silencing variants in GM12878 (Fig. 2d), 106 allelic enhancing and 179 allelic silencing variants in MS-1 (Fig. 2e), and 28 allelic enhancing and 110 allelic silencing variants in MS-2 (Fig. 2f). Altogether, we identified a total of 150 MS risk variants with allelic enhancing activity (13% of enhancer variants) (Fig. 2g) and 286 MS risk variants with allelic silencing activity (13.5% of silencer variants) (Fig. 2h) across the three cell lines (Supplementary Table 7).

**Fig. 3.**
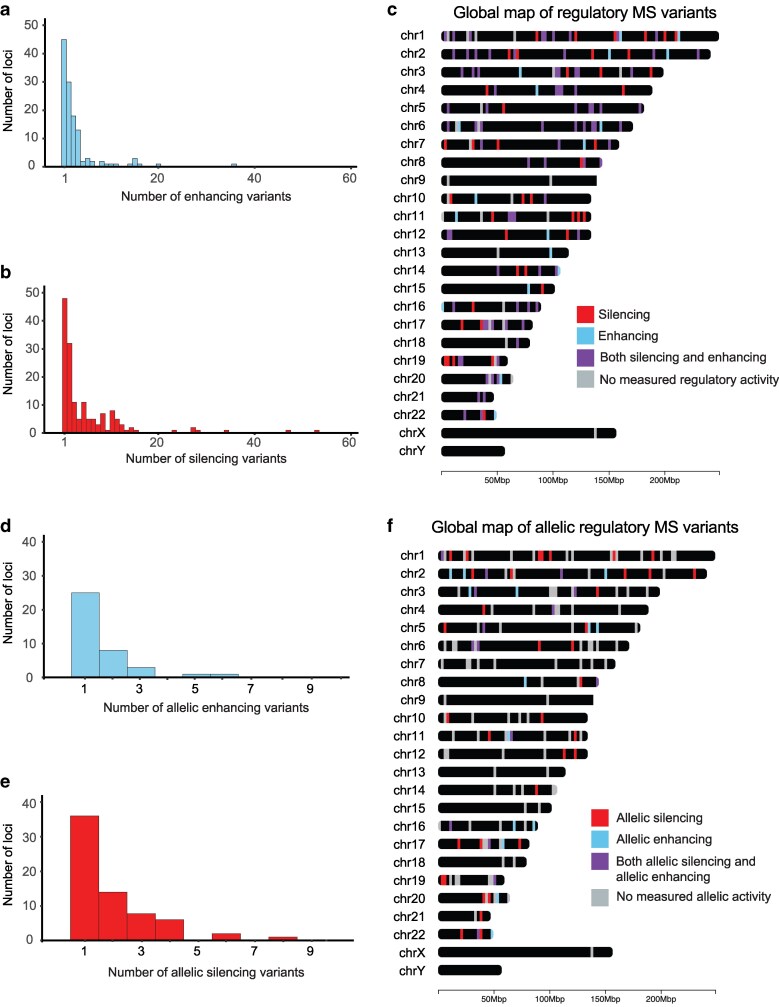
Distribution of regulatory and allelic regulatory variants across independent MS risk loci. The distributions of the number of MS risk variants with enhancing (a) and silencing (b) activity within each independent MS risk locus are shown as a histogram. Loci with zero enhancing or silencing variants are excluded. c) Chromomap depicting the location of regulatory variants with enhancing (blue) and silencing (red) activity across MS risk loci. Loci containing both variants with enhancing and silencing activity are shown in purple. The distribution of the number of MS risk variants with allelic enhancing (d) and allelic silencing (e) activity within independent MS risk loci are shown as histograms. Loci with zero allelic enhancing or allelic silencing variants are excluded. f) Chromomap depicting the location of regulatory variants with enhancing (blue) and silencing (red) activity across MS risk loci. Loci containing both variants with enhancing and silencing activity are shown in purple.

In total, 181 of the 217 tested independent MS genetic risk loci displayed regulatory activity, with each locus having between 1 and 37 variants with enhancing activity (median of 2) (Fig. 3a) and between one and 54 variants with silencing activity (median of 2) (Fig. 3b). Notably, 99 of these 181 risk loci contained both variants with enhancing activity and variants with silencing activity (Fig. 3c). Similar to the findings of other disease-specific MPRA studies (Choi et al. 2020; Lu et al. 2021; Abell et al. 2022; McAfee et al. 2023; Shook et al. 2024), some independent risk loci also contained multiple variants with allelic regulatory activity. While most independent loci had zero or one variant with either allelic enhancing or allelic silencing activity (Fig. 3, d and e), some loci had up to 6 allelic enhancing or 8 allelic silencing variants, and 21 loci contained both allelic enhancing and allelic silencing variants (Fig. 3f). Altogether, we identified allelic regulatory activity at 83 of the 217 tested independent MS risk loci (38%). We further identified allelic regulatory variants at 114 MS risk loci with suggestive significance (Supplementary Table 7). There is a high overlap of MS risk variants, genes, and pathways with other autoimmune diseases, reflecting robust pleiotropy with other diseases involving breaks in immunological tolerance (International Multiple Sclerosis Genetics et al. 2011). As expected, many of our allelic regulatory variants are located in risk haplotypes shared with other autoimmune diseases, including systemic lupus erythematosus (SLE), Crohn's disease, and rheumatoid arthritis (Supplementary Table 8).

### Assessment of transcription factor binding and gene expression at MS loci with allelic regulatory activity

In order to elucidate the gene regulatory mechanisms that might be impacted by these allelic enhancer and silencer regulatory variants, we identified functional genomics features enriched within allelic regulatory variants relative to all variants contained in the MPRA using the RELI algorithm (Harley et al. 2018). These analyses revealed that allelic enhancers are enriched for ChIP-seq peaks in B cell lines for regulatory proteins associated with the transcription preinitiation complex, including EP300 and POLR2A, as well as for potent activators RELA (an NFkB subunit) and VDR. In addition, allelic silencers are highly enriched for ChIP-seq peaks for chromatin remodelers such as CBX5 (a component of heterochromatin that leads to epigenetic-based repression) as well as transcription factors important for B lymphocyte differentiation, such as PAX5 and EGR1, which both play established repressive roles in B cells (Dinkel et al. 1997; Eberhard et al. 2000; Inagaki et al. 2016) (Supplementary Table 9).

**Fig. 4.**
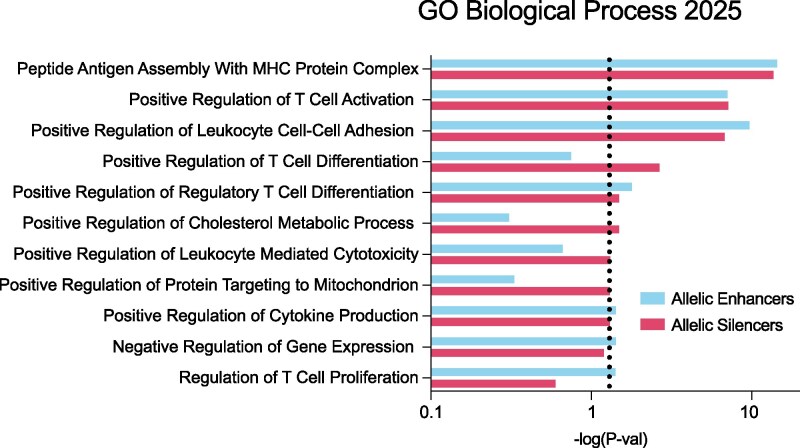
Genes connected to MS allelic regulatory variants are enriched for disease-relevant biological pathways. Genes that are eQTLs in EBV-transformed B cells for variants with allelic enhancing (blue, top) and silencing (red, bottom) activity were identified using the eQTL Catalogue. Enriched biological processes are shown. Only pathways with five or more gene overlaps are shown. Redundant pathways (with largely overlapping genes) were removed. See Supplementary Table 11 for all results.

We next sought to connect allelic regulatory variants to putative gene targets. To this end, allelic enhancing and silencing variants were annotated with all SNP-gene associations consistent with the direction of allelic effect using EBV-transformed B cell data obtained from the eQTL Catalogue (Supplementary Table 10). A total of 94 and 207 allelic regulatory variants were annotated as eQTLs for enhancing and silencing variants, respectively. Genes connected to MS risk variants with both allelic enhancing and allelic silencing activity were significantly enriched for pathways related to antigen presentation and B cell–T cell interactions (Fig. 4, Supplementary Table 11). Genes connected to allelic silencing MS risk variants were uniquely enriched for pathways related to leukocyte-mediated cytotoxicity (Fig. 4).

## Discussion

In this study, we developed a MPRA to investigate the allelic regulatory activity of genetic variants associated with MS. This approach allowed us to systematically assess the enhancing and silencing activity of 14,275 variants across 217 independent established MS risk loci and an additional 411 loci with suggestive significance, providing valuable insights into the genetic mechanisms underlying this complex disease.

In total, our study identifies 150 allelic enhancing variants and 286 allelic silencing variants, representing 83 independent established MS risk loci. All of these variants exhibit genotype-dependent regulatory activity, highlighting the importance of disease-associated genetic variation in modulating gene expression in MS. Notably, our results demonstrate that a significant proportion of MS risk loci contains both enhancing and silencing variants, suggesting that these loci may have complex regulatory landscapes that contribute to disease pathogenesis.

Allelic variants can significantly impact gene expression, thereby contributing to the etiology of MS. The study of allelic gene expression, particularly through expression quantitative trait loci (eQTL) analysis, provides valuable insights into how these variants influence gene activity. Our integration of allelic MPRA results with eQTL results identifies specific enriched pathways involving genes that are known to contribute to dysregulated B-T cell interactions, cytokine production, and antigen presentation (Fig. 4). Each of these pathways has previously been shown to play important roles in the development and pathogenesis of MS (Chastain et al. 2011; Legroux and Arbour 2015; He et al. 2018; Nuzziello et al. 2018; Liu et al. 2022; Manna et al. 2024). These findings suggest that MS-associated allelic regulatory variants may have broad implications for B cell function and disease susceptibility. Taken together, our study supports a model in which allelic perturbations to gene expression within these pathways in B cells collectively contribute to the genetic basis of MS.

The use of EBV-transformed B cells, including patient-derived cell lines, was a crucial aspect of our experimental design. B cells play a central role in MS and are a primary therapeutic target (van Langelaar et al. 2020). By utilizing B cells from patients with MS, we aimed to capture disease-relevant regulatory activity that may not be present in commercially available cell lines. EBV is the leading cause of MS (Bjornevik et al. 2022) and several lines of evidence suggest that EBV interacts with MS genetic risk variants to drive disease (Harley et al. 2018; Afrasiabi et al. 2020; Hong et al. 2021; Jacobs et al. 2021). Therefore, EBV-transformed B cells are a highly relevant model system for studying gene x environment interactions in the context of MS genetic risk variants. While the allelic results were largely shared across B cell lines, we identified distinct allelic regulatory variants in the patient-derived B cell lines, underscoring the importance of studying disease-specific cellular contexts.

Due to the complex role of genetic risk factors in MS, we aimed to simultaneously assess both enhancing and silencing activity of variants in the context of disease-relevant cell types. Our study highlights the utility of the RSV promoter in detecting variants with silencing activity, which have traditionally been understudied despite increasing evidence of their contributions to disease (Della Rosa and Spivakov 2020; Huang and Ovcharenko 2024). The stronger basal activity of the RSV promoter allowed us to identify variants with silencing activity that could not have been identified using standard minimal promoters. This approach provides a more comprehensive understanding of the regulatory mechanisms at play in MS.

Despite our strong results, our study has several limitations. First, like most studies to date, the MPRA presented relies on transient transfections, so the library does not adopt the native chromatin context of these variants. Additionally, our study exclusively used B cell lines, which limits the generalizability of the findings to other cell types that may also play crucial roles in the etiology of MS. Furthermore, we only examined 150 bp regions, and a broader context could reveal more disease-relevant biology. The use of the RSV promoter allows for the detection of both enhancing and silencing variants; however, this strategy identifies far more silencing variants than enhancing ones. Future MPRA libraries employing multiple categories of promoters will likely uncover additional genetic variants with allelic enhancing and silencing activity. Finally, MPRA does not identify target genes, and eQTL assessment could miss genes that are only important in specific contexts, together underscoring the need for complementary approaches to fully understand the functional impact of disease-associated genetic variants.

While MPRAs are a powerful approach for uncovering putative regulatory variants, they often yield large numbers of candidates, making downstream functional validation challenging. To address this, it is essential to develop a systematic strategy for prioritizing the most likely functional variants. In our study, we use publicly available data to prioritize MPRA-derived allelic regulatory variants. In particular, by connecting allelic regulatory variants to genes with genotype-dependent expression levels in the same cell type (as measured through eQTLs), we implicate genes in disease-relevant pathways such as antigen presentation and cytokine production. In addition, comparison to ChIP-seq experiments performed in the same cell type enables us to systematically identify features enriched within allelic regulatory variants relative to all variants tested in the MPRA, using our RELI algorithm (Harley et al. 2018). These analyses revealed that allelic enhancers are strongly enriched for ChIP-seq peaks for regulatory proteins such as EP300, POLR2A, RELA (an NFkB subunit), and VDR. In contrast, allelic silencers are highly enriched for ChIP-seq peaks for chromatin remodelers such as CBX5 as well as transcription factors PAX5 and EGR1, which both play established repressive roles in B cells (Dinkel et al. 1997; Eberhard et al. 2000; Inagaki et al. 2016) (Supplementary Table 9). Future studies employing genome editing to validate these allelic regulatory variants for their impact on protein binding, gene expression, and cell behavior will lead toward a comprehensive understanding of how individual genetic variants contribute to MS disease mechanisms.

In conclusion, our study demonstrates the power of MPRAs to uncover allelic regulatory activity for 83 MS-associated genetic variants across 217 tested independent risk loci. These allelic enhancing and allelic silencing variants provide a valuable resource for understanding the genetic basis of MS. Future research should focus on elucidating the functional consequences of these variants and exploring their potential as therapeutic targets.

## Supplementary Material

jkaf192_Supplementary_Data

## Data Availability

All sequencing data are available in the GEO database under accession number GSE293036. Full datasets and processed results are provided in the Supplementary datasets. All other data are available from the corresponding authors upon request. IRB #2017-0430. Source code, with full documentation and examples, are freely available under Version 3 of the GNU General Public License on the WeirauchLab GitHub page: https://github.com/WeirauchLab/mpraprofiler. Additional scripts will be provided upon request. Supplemental material available at G3 online.

## References

[jkaf192-B1] Abell NS et al 2022. Multiple causal variants underlie genetic associations in humans. Science. 375:1247–1254. 10.1126/science.abj5117. 35298243 PMC9725108

[jkaf192-B2] Abrahamyan S et al 2020. Complete Epstein-Barr virus seropositivity in a large cohort of patients with early multiple sclerosis. J Neurol Neurosurg Psychiatry. 91:681–686. 10.1136/jnnp-2020-322941. 32371533 PMC7361012

[jkaf192-B3] Afrasiabi A, Parnell GP, Swaminathan S, Stewart GJ, Booth DR. 2020. The interaction of multiple sclerosis risk loci with Epstein-Barr virus phenotypes implicates the virus in pathogenesis. Sci Rep. 10:193. 10.1038/s41598-019-55850-z. 31932685 PMC6957475

[jkaf192-B4] Ahn JJ, Abu-Rub M, Miller RH. 2021. B cells in neuroinflammation: new perspectives and mechanistic insights. Cells. 10:1605. 10.3390/cells10071605. 34206848 PMC8305155

[jkaf192-B5] Bjornevik K et al 2022. Longitudinal analysis reveals high prevalence of Epstein-Barr virus associated with multiple sclerosis. Science. 375:296–301. 10.1126/science.abj8222. 35025605

[jkaf192-B6] Bolger AM, Lohse M, Usadel B. 2014. Trimmomatic: a flexible trimmer for illumina sequence data. Bioinformatics. 30:2114–2120. 10.1093/bioinformatics/btu170. 24695404 PMC4103590

[jkaf192-B7] Buniello A et al 2019. The NHGRI-EBI GWAS catalog of published genome-wide association studies, targeted arrays and summary statistics 2019. Nucleic Acids Res. 47:D1005–D1012. 10.1093/nar/gky1120. 30445434 PMC6323933

[jkaf192-B8] Byrska-Bishop M et al 2022. High-coverage whole-genome sequencing of the expanded 1000 genomes project cohort including 602 trios. Cell. 185:3426–3440.e3419. 10.1016/j.cell.2022.08.004. 36055201 PMC9439720

[jkaf192-B9] Chastain EM, Duncan DS, Rodgers JM, Miller SD. 2011. The role of antigen presenting cells in multiple sclerosis. Biochim Biophys Acta. 1812:265–274. 10.1016/j.bbadis.2010.07.008. 20637861 PMC2970677

[jkaf192-B10] Chen EY et al 2013. Enrichr: interactive and collaborative html5 gene list enrichment analysis tool. BMC Bioinformatics. 14:128. 10.1186/1471-2105-14-128. 23586463 PMC3637064

[jkaf192-B11] Choi J et al 2020. Massively parallel reporter assays of melanoma risk variants identify mx2 as a gene promoting melanoma. Nat Commun. 11:2718. 10.1038/s41467-020-16590-1. 32483191 PMC7264232

[jkaf192-B12] Della Rosa M, Spivakov M. 2020. Silencers in the spotlight. Nat Genet. 52:244–245. 10.1038/s41588-020-0583-8. 32094910

[jkaf192-B13] DeLorenze GN et al 2006. Epstein-Barr virus and multiple sclerosis: evidence of association from a prospective study with long-term follow-up. Arch Neurol. 63:839–844. 10.1001/archneur.63.6.noc50328. 16606758

[jkaf192-B14] DeMarshall C et al 2017. Autoantibodies as diagnostic biomarkers for the detection and subtyping of multiple sclerosis. J Neuroimmunol. 309:51–57. 10.1016/j.jneuroim.2017.05.010. 28601288

[jkaf192-B15] Dinkel A et al 1997. Transcription factor Egr-1 activity down-regulates Fas and CD23 expression in B cells. J Immunol. 159:2678–2684. 10.4049/jimmunol.159.6.2678. 9300687

[jkaf192-B16] Eberhard D, Jimenez G, Heavey B, Busslinger M. 2000. Transcriptional repression by pax5 (bsap) through interaction with corepressors of the groucho family. EMBO J. 19:2292–2303. 10.1093/emboj/19.10.2292. 10811620 PMC384353

[jkaf192-B17] Filippi M et al 2018. Multiple sclerosis. Nat Rev Dis Primers. 4:43. 10.1038/s41572-018-0041-4. 30410033

[jkaf192-B18] Harley JB et al 2018. Transcription factors operate across disease loci, with ebna2 implicated in autoimmunity. Nat Genet. 50:699–707. 10.1038/s41588-018-0102-3. 29662164 PMC6022759

[jkaf192-B19] He H, Hu Z, Xiao H, Zhou F, Yang B. 2018. The tale of histone modifications and its role in multiple sclerosis. Hum Genomics. 12:31. 10.1186/s40246-018-0163-5. 29933755 PMC6013900

[jkaf192-B20] Hollenbach JA, Oksenberg JR. 2015. The immunogenetics of multiple sclerosis: a comprehensive review. J Autoimmun. 64:13–25. 10.1016/j.jaut.2015.06.010. 26142251 PMC4687745

[jkaf192-B21] Hong T et al 2021. Epstein-Barr virus nuclear antigen 2 extensively rewires the human chromatin landscape at autoimmune risk loci. Genome Res. 31:2185–2198. 10.1101/gr.264705.120. 34799401 PMC8647835

[jkaf192-B22] Huang D, Ovcharenko I. 2024. The contribution of silencer variants to human diseases. Genome Biol. 25:184. 10.1186/s13059-024-03328-1. 38978133 PMC11232194

[jkaf192-B23] Inagaki Y et al 2016. Pax5 tyrosine phosphorylation by syk co-operatively functions with its serine phosphorylation to cancel the pax5-dependent repression of blimp1: a mechanism for antigen-triggered plasma cell differentiation. Biochem Biophys Res Commun. 475:176–181. 10.1016/j.bbrc.2016.05.067. 27181361

[jkaf192-B24] International Multiple Sclerosis Genetics Consortium . 2019. Multiple sclerosis genomic map implicates peripheral immune cells and microglia in susceptibility. Science. 365:6460. 10.1126/science.aav7188. PMC7241648 31604244

[jkaf192-B25] International Multiple Sclerosis Genetics Consortium et al 2011. Genetic risk and a primary role for cell-mediated immune mechanisms in multiple sclerosis. Nature. 476:214–219. 10.1038/nature10251. 21833088 PMC3182531

[jkaf192-B26] Jacobs BM et al 2021. Gene-environment interactions in multiple sclerosis: a UK biobank study. Neurol Neuroimmunol Neuroinflamm. 8:e1007. 10.1212/NXI.0000000000001007. 34049995 PMC8192056

[jkaf192-B27] Kerimov N et al 2021. A compendium of uniformly processed human gene expression and splicing quantitative trait loci. Nat Genet. 53:1290–1299. 10.1038/s41588-021-00924-w. 34493866 PMC8423625

[jkaf192-B28] Kuleshov MV et al 2016. Enrichr: a comprehensive gene set enrichment analysis web server 2016 update. Nucleic Acids Res. 44:W90–W97. 10.1093/nar/gkw377. 27141961 PMC4987924

[jkaf192-B29] Langmead B, Salzberg SL. 2012. Fast gapped-read alignment with bowtie 2. Nat Methods. 9:357–359. 10.1038/nmeth.1923. 22388286 PMC3322381

[jkaf192-B30] Lanz TV et al 2022. Clonally expanded b cells in multiple sclerosis bind ebv ebna1 and glialcam. Nature. 603:321–327. 10.1038/s41586-022-04432-7. 35073561 PMC9382663

[jkaf192-B31] Legroux L, Arbour N. 2015. Multiple sclerosis and t lymphocytes: an entangled story. J Neuroimmune Pharmacol. 10:528–546. 10.1007/s11481-015-9614-0. 25946987 PMC5052065

[jkaf192-B32] Liu R et al 2022. Autoreactive lymphocytes in multiple sclerosis: pathogenesis and treatment target. Front Immunol. 13:996469. 10.3389/fimmu.2022.996469. 36211343 PMC9539795

[jkaf192-B33] Love MI, Huber W, Anders S. 2014. Moderated estimation of fold change and dispersion for RNA-Seq data with deseq2. Genome Biol. 15:550. 10.1186/s13059-014-0550-8. 25516281 PMC4302049

[jkaf192-B34] Lu X et al 2021. Global discovery of lupus genetic risk variant allelic enhancer activity. Nat Commun. 12:1611. 10.1038/s41467-021-21854-5. 33712590 PMC7955039

[jkaf192-B35] Ma Q et al 2023. Integration of epigenetic and genetic profiles identifies multiple sclerosis disease-critical cell types and genes. Commun Biol. 6:342. 10.1038/s42003-023-04713-5. 36997638 PMC10063586

[jkaf192-B36] Manna I, De Benedittis S, Porro D. 2024. A comprehensive examination of the role of epigenetic factors in multiple sclerosis. Int J Mol Sci. 25:8921. 10.3390/ijms25168921. 39201606 PMC11355011

[jkaf192-B37] Maurano MT et al 2012. Systematic localization of common disease-associated variation in regulatory DNA. Science. 337:1190–1195. 10.1126/science.1222794. 22955828 PMC3771521

[jkaf192-B38] McAfee JC et al 2023. Systematic investigation of allelic regulatory activity of schizophrenia-associated common variants. Cell Genom. 3:100404. 10.1016/j.xgen.2023.100404. 37868037 PMC10589626

[jkaf192-B39] Nuzziello N et al 2018. Investigating the role of microrna and transcription factor co-regulatory networks in multiple sclerosis pathogenesis. Int J Mol Sci. 19:3652. 10.3390/ijms19113652. 30463275 PMC6274935

[jkaf192-B40] Olsson T, Barcellos LF, Alfredsson L. 2017. Interactions between genetic, lifestyle and environmental risk factors for multiple sclerosis. Nat Rev Neurol. 13:25–36. 10.1038/nrneurol.2016.187. 27934854

[jkaf192-B41] Purcell S et al 2007. Plink: a tool set for whole-genome association and population-based linkage analyses. Am J Hum Genet. 81:559–575. 10.1086/519795. 17701901 PMC1950838

[jkaf192-B42] Putscher E et al 2022. Genetic risk variants for multiple sclerosis are linked to differences in alternative pre-mRNA splicing. Front Immunol. 13:931831. 10.3389/fimmu.2022.931831. 36405756 PMC9670805

[jkaf192-B43] Rastogi I et al 2022. Role of B cells as antigen presenting cells. Front Immunol. 13:954936. 10.3389/fimmu.2022.954936. 36159874 PMC9493130

[jkaf192-B44] Robinson WH, Steinman L. 2022. Epstein-Barr virus and multiple sclerosis. Science. 375:264–265. 10.1126/science.abm7930. 35025606

[jkaf192-B45] Shook MS et al 2024. Systematic identification of genotype-dependent enhancer variants in eosinophilic esophagitis. Am J Hum Genet. 111:280–294. 10.1016/j.ajhg.2023.12.008. 38183988 PMC10870143

[jkaf192-B46] Stathopoulos P, Dalakas MC. 2022. Evolution of anti-B cell therapeutics in autoimmune neurological diseases. Neurotherapeutics. 19:691–710. 10.1007/s13311-022-01196-w. 35182380 PMC9294112

[jkaf192-B47] Tewhey R et al 2016. Direct identification of hundreds of expression-modulating variants using a multiplexed reporter assay. Cell. 165:1519–1529. 10.1016/j.cell.2016.04.027. 27259153 PMC4957403

[jkaf192-B48] Ulirsch JC et al 2016. Systematic functional dissection of common genetic variation affecting red blood cell traits. Cell. 165:1530–1545. 10.1016/j.cell.2016.04.048. 27259154 PMC4893171

[jkaf192-B49] van Langelaar J, Rijvers L, Smolders J, van Luijn MM. 2020. B and t cells driving multiple sclerosis: identity, mechanisms and potential triggers. Front Immunol. 11:760. 10.3389/fimmu.2020.00760. 32457742 PMC7225320

[jkaf192-B50] Viel K et al 2024. Shared and distinct interactions of type 1 and type 2 epstein-barr nuclear antigen 2 with the human genome. BMC Genomics. 25:273. 10.1186/s12864-024-10183-8. 38475709 PMC10935964

[jkaf192-B51] Walton C et al 2020. Rising prevalence of multiple sclerosis worldwide: insights from the atlas of ms, third edition. Mult Scler. 26:1816–1821. 10.1177/1352458520970841. 33174475 PMC7720355

