# Supplementary material for: Genome-wide discovery of multiple sclerosis genetic risk variant allelic regulatory activity
Source: G3 (Bethesda). 2025 Aug 21;15(11):jkaf192. doi: 10.1093/g3journal/jkaf192 (PMC12608076; doi:10.1093/g3journal/jkaf192)
Supplement: jkaf192_Supplementary_Data [file jkaf192_supplementary_data.zip › Supplementary_Figure_3_G3-2025-406100.pdf]

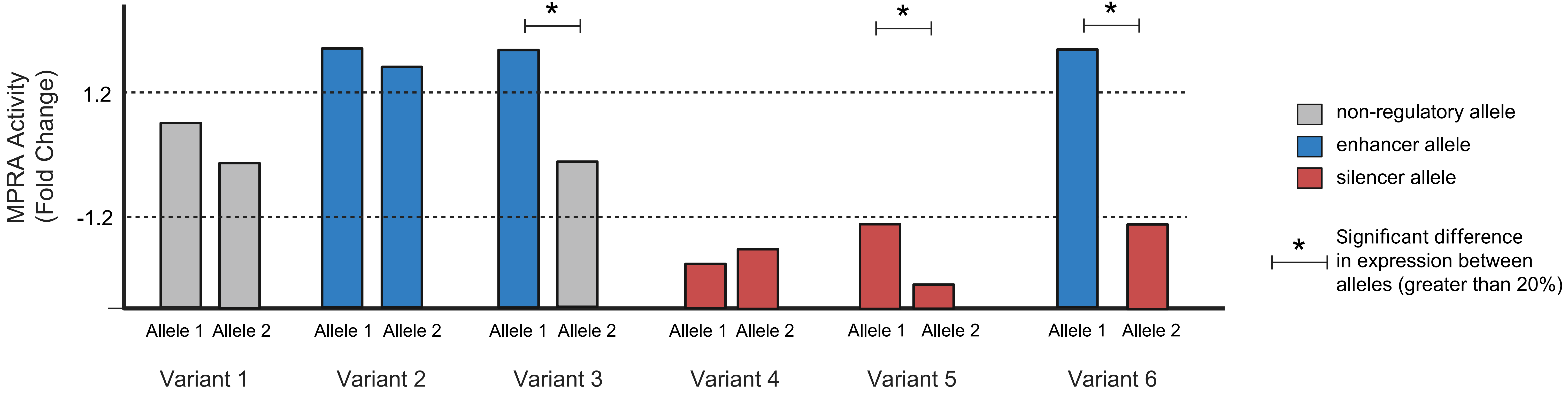

	Variant 1	Variant 2	Variant 3	Variant 4	Variant 5	Variant 6
Enhancer?	N	Y	Y	N	N	Y
Allelic enhancer?	N	N	Y	N	N	Y
Silencer?	N	N	N	Y	Y	Y
Allelic silencer?	N	N	N	N	Y	Y
