# Supplementary material for: Genome-wide discovery of multiple sclerosis genetic risk variant allelic regulatory activity
Source: G3 (Bethesda). 2025 Aug 21;15(11):jkaf192. doi: 10.1093/g3journal/jkaf192 (PMC12608076; doi:10.1093/g3journal/jkaf192)
Supplement: jkaf192_Supplementary_Data [file jkaf192_supplementary_data.zip › Supplementary_Material_Legends_G3-2025-406100.docx]

**Supplementary Material Legends**

**Supplementary Figures**

**Supplementary Figure 1. Histogram showing the distribution of unique barcodes associated with each oligo (allele of an MS risk variant) in the plasmid transfection library**.

**Supplementary Figure 2. Quality assessment of MPRA experiments**. a. Principal component analysis (PCA) depicting 3 plasmid controls and 5 replicates for each of the 3 transfected EBV-transformed B cell lines. b-d. Correlation between normalized barcode counts of regulatory variants in three replicates of the plasmid library and five replicates of GM12878 (b), MS-1 (c), and MS-2 (d). Pearson’s r for each pairwise comparison is shown.

**Supplementary Figure 3. Schematic for the identification of regulatory variants and allelic regulatory variants in the MPRA.** Alleles are classified based on MPRA activity, which is measured as the fold change difference in expression relative to plasmid controls. Alleles showing a significant increase in expression (adjusted p-value < 0.05) above a 1.2-fold threshold are classified as enhancers (blue bars), while those showing a significant decrease below a -1.2-fold threshold are classified as silencers (red bars). Alleles without significant MPRA activity are shown in gray. Enhancer variants are defined as genetic variants with at least one allele above the enhancer cutoff (variants 2, 3, and 6 in this schematic). Allelic enhancer variants are enhancer variants with a significant difference of at least 20% between alleles (adjusted p-value < 0.05, Student’s t test, indicated with asterisks). Silencer variants are defined as genetic variants with at least one allele below the silencer cutoff (variants 4, 5, and 6 in this schematic). Allelic silencer variants are silencer variants with a significant difference of at least 20% between alleles (adjusted p-value < 0.05, Student’s t test, indicated with asterisks). Figure adapted from *Shook et al 2024*.

**Supplementary Figure 4. Shared and unique regulatory variants**. a. Venn diagram of shared and unique enhancer variants in GM12878, MS-1, and MS-2. b. Venn diagram of shared and unique silencer variants in GM12878, MS-1, and MS-2.

**Supplementary Figure 5. Enrichment of histone marks in EBV-transformed B cells at enhancer and silencer variants.** The RELI algorithm (see Methods) was used to identify enrichment of histone marks from public ChIP-seq datasets for (a) enhancing variants and (b) silencing variants. Complete results can be found in Supplementary Table 6.

**Supplementary Tables**

**Supplementary Table 1.** Tag variants included in the MPRA

**Supplementary Table 2.** LD expanded MS variants

**Supplementary Table 3.** MS MPRA variant sequences ordered from Twist

**Supplementary Table 4.** List of primers used to build the MPRA library

**Supplementary Table 5.** Regulatory variant identification

**Supplementary Table 6.** Histone mark enrichment analysis for enhancing and silencing variants

**Supplementary Table 7.** Allelic regulatory variant identification

**Supplementary Table 8.** Pleiotropy of allelic regulatory variants with other disease risk haplotypes

**Supplementary Table 9.** Transcription factor enrichment analysis for allelic silencer variants and allelic enhancer variants

**Supplementary Table 10.** eQTLs identified at allelic regulatory variants

**Supplementary Table 11.** Pathway analysis for allelic silencer and allelic enhancer putative gene targets
