# Supplementary figures and images for: Genome-wide discovery of multiple sclerosis genetic risk variant allelic regulatory activity
Source: G3 (Bethesda). 2025 Aug 21;15(11):jkaf192. doi: 10.1093/g3journal/jkaf192 (PMC12608076; doi:10.1093/g3journal/jkaf192)

Distribution of unique barcodes associated with each oligo in the transfection library

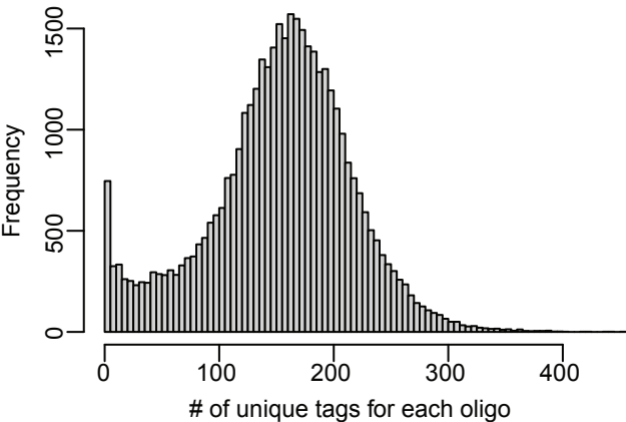

Supplement: jkaf192_Supplementary_Data [file jkaf192_supplementary_data.zip › Supplementary_Figure_1_G3-2025-406100.pdf]

b.

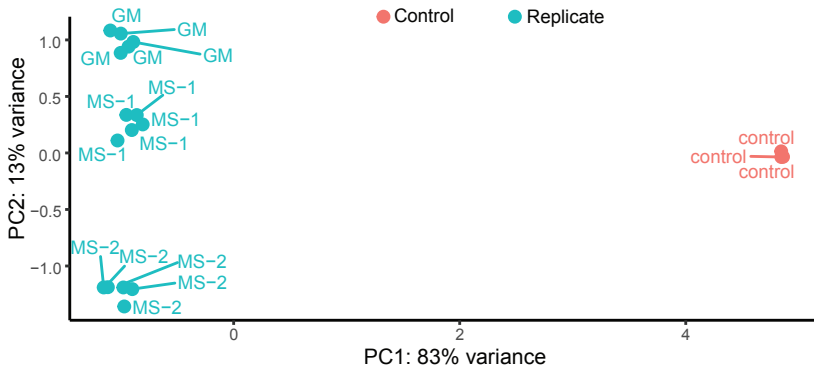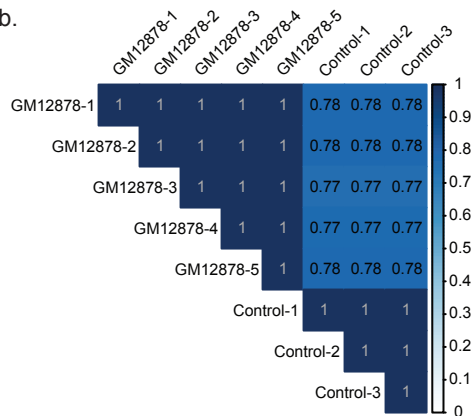

C.

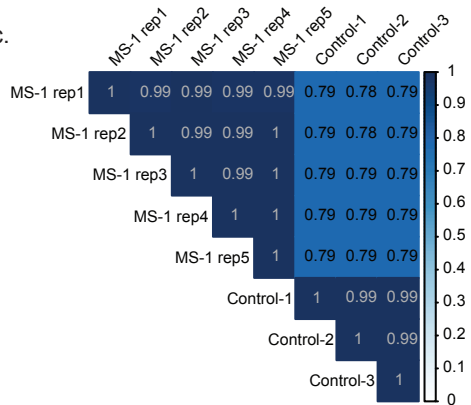

d.

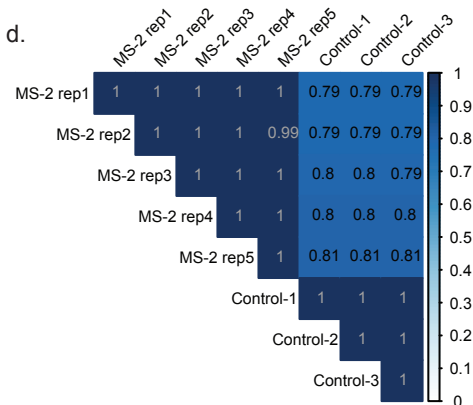

Supplement: jkaf192_Supplementary_Data [file jkaf192_supplementary_data.zip › Supplementary_Figure_2_G3-2025-406100.pdf]

a. Shared and unique enhancer variants

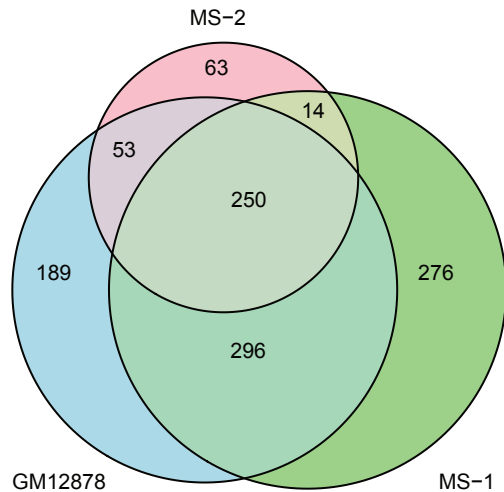

b. Shared and unique silencer variants

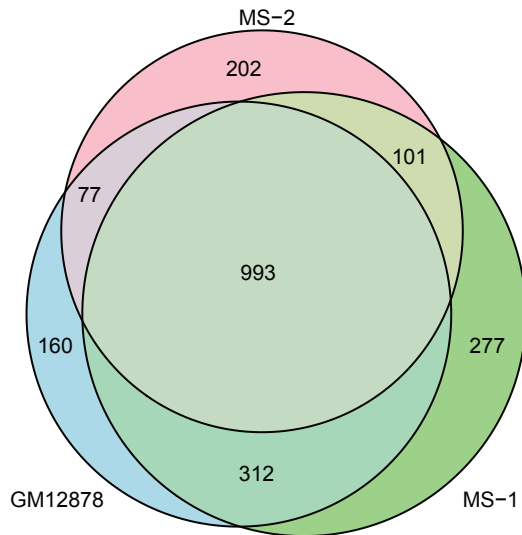

Supplement: jkaf192_Supplementary_Data [file jkaf192_supplementary_data.zip › Supplementary_Figure_4_G3-2025-406100.pdf]

a.

## Enhancer variant histone mark enrichment

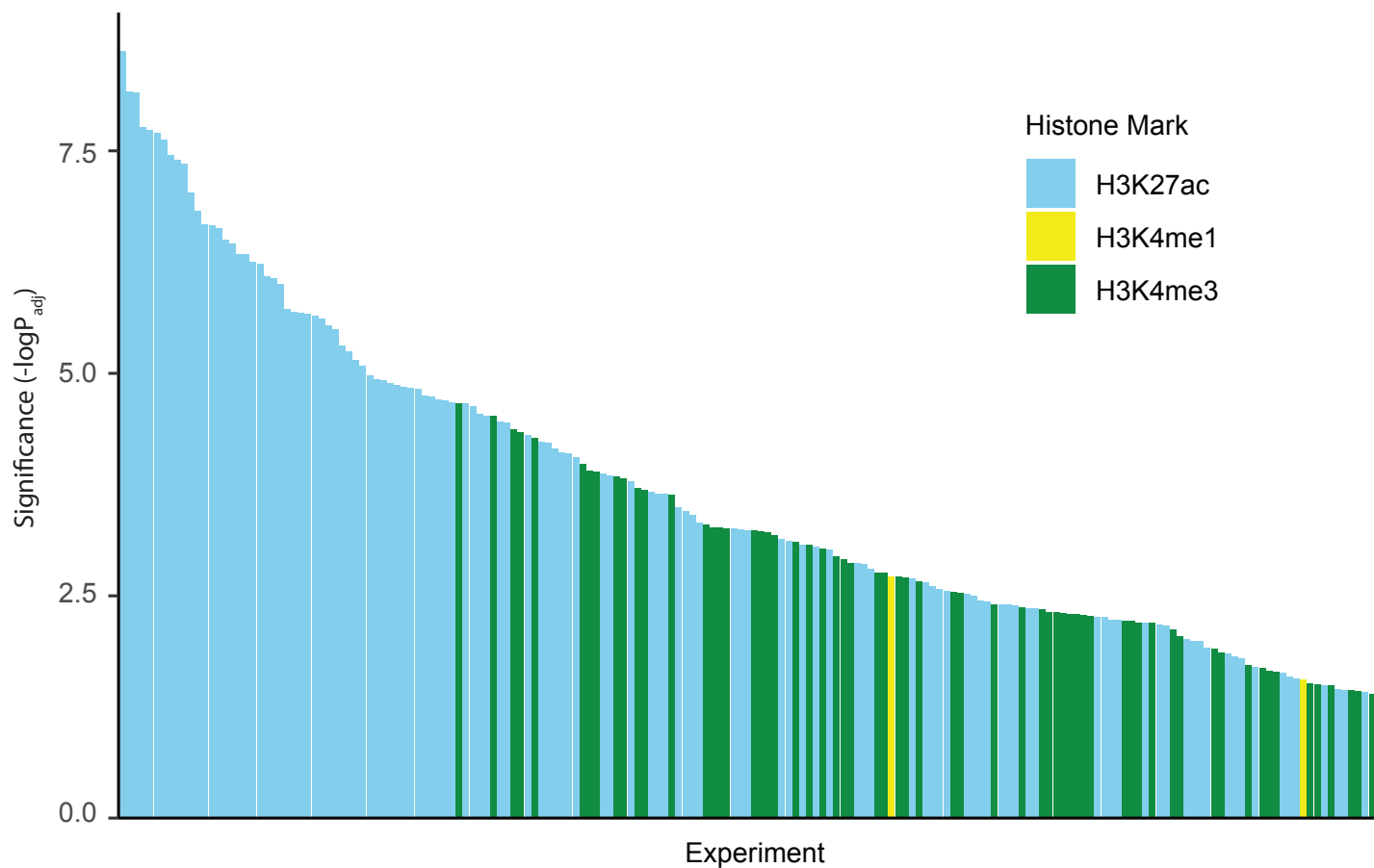

b.

## Silencer variant histone mark enrichment

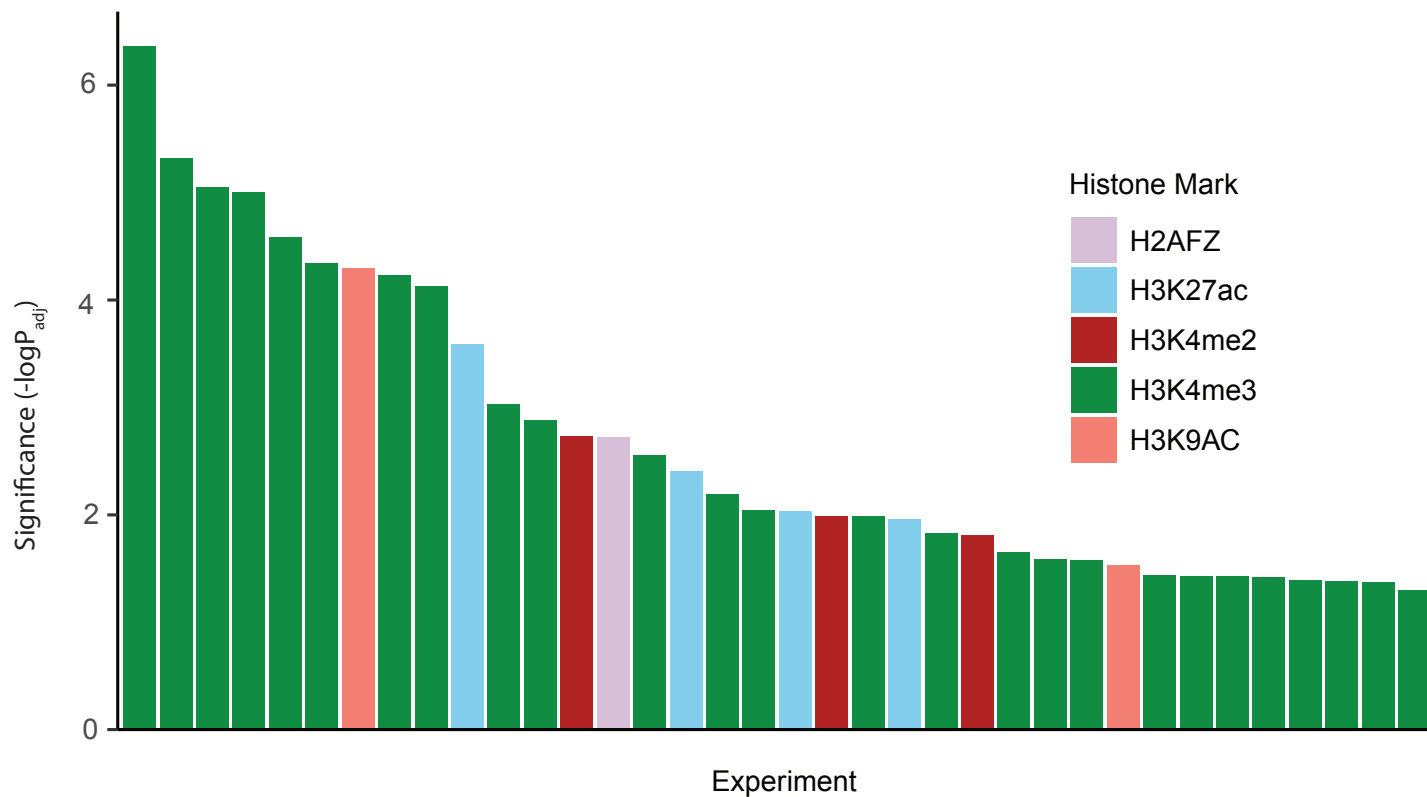

Supplement: jkaf192_Supplementary_Data [file jkaf192_supplementary_data.zip › Supplementary_Figure_5_G3-2025-406100.pdf]
